# Oncogenes in high grade serous adenocarcinoma of the ovary

**DOI:** 10.18632/genesandcancer.206

**Published:** 2020-11-11

**Authors:** Pacharla Manasa, Chirukandath Sidhanth, Syama Krishnapriya, Sekar Vasudevan, Trivadi S. Ganesan

**Affiliations:** ^1^ Laboratory for Cancer Biology, Department of Medical Oncology and Clinical Research Cancer Institute (WIA), Chennai, India

**Keywords:** oncogenes, ovarian cancer, high grade serous adenocarcinoma of the ovary, TCGA, copy number alterations

## Abstract

High grade serous ovarian cancer is characterized by relatively few mutations occurring at low frequency, except in TP53. However other genetic aberrations such as copy number variation alter numerous oncogenes and tumor suppressor genes. Oncogenes are positive regulators of tumorigenesis and play a critical role in cancer cell growth, proliferation, and survival. Accumulating evidence suggests that they are crucial for the development and the progression of high grade serous ovarian carcinoma (HGSOC). Though many oncogenes have been identified, no successful inhibitors targeting these molecules and their associated pathways are available. This review discusses oncogenes that have been identified recently in HGSOC using different screening strategies. All the genes discussed in this review have been functionally characterized both *in vitro* and *in vivo* and some of them are able to transform immortalized ovarian surface epithelial and fallopian tube cells upon overexpression. However, it is necessary to delineate the molecular pathways affected by these oncogenes for the development of therapeutic strategies.

## INTRODUCTION

### 

Ovarian cancer represents the most common cause of death globally due to gynecological cancer. In India, in the year 2012, approximately 26,834 women were diagnosed with ovarian cancer, of which 19,549 died [1]. Most of the patients are diagnosed at an advanced stage, resulting in reduced survival. The abdominal cavity, in particular, the peritoneum is the most common site of metastases. The current standard treatment for ovarian cancer is a combination of chemotherapy and surgery. Despite this, the five-year survival of patients with advanced stage has remained at around 30% [2]. Hence, there is a critical need for understanding the pathogenesis of the disease to identify additional therapeutic targets in ovarian cancer.

Ovarian cancer is a complex and heterogeneous disease. It is classified into epithelial tumors, sex cord-stromal tumors, and germ cell tumors based on morphology. More than 90% of ovarian tumors are epithelial in origin. Based on the histology and molecular alterations, the epithelial tumors are subdivided into four main subtypes: serous, endometrioid, clear cell, and mucinous. Among these subtypes, HGSOC is the most common contributing to 80% of mortality from ovarian cancer [3]. The precise cellular origin of HGSOC is unclear. Traditionally these tumors were proposed to arise from the ovarian surface epithelium (OSE) especially from the cortical inclusion cyst (CIC). This was supported by the ‘incessant ovulation’ hypothesis and through several genetically modified mouse models. However recent evidence demonstrates that HGSOC can also arise from the fimbrial epithelium of the fallopian tube. Identification of invasive lesions in the fimbriae of fallopian tubes from the prophylactic salpingo-oophorectomies of women at high risk of developing ovarian cancer due to germline mutations of BRCA1 and BRCA2 supported this hypothesis. Later, it was demonstrated that the precursor lesions, serous tubal intraepithelial carcinoma (STIC), were found in the fimbriae of women with sporadic HGSOC (21-59%) and with hereditary HGSOC (3-31%). These observations were confirmed using murine models, where fallopian tube epithelial (FTE) cells with different combinations of genetic mutations in TP53, BRCA1/2, PTEN, NF1, and RB1 when injected into the mouse, developed STIC and progressed to HGSOC. Besides, it was also shown that the FTE cells share molecular profiles similar to HGSOC, than OSE. However, the hypothesis that the origin of HGSOC from FTE cells has the following limitations. Though the presence of STIC was identified in a subset of tumors, the cell of origin in the remainder is elusive. Furthermore, the development of HGSOC even after salpingectomy has been observed both in mice as well as in patients [4]. Thus it is possible that both these sites may have the potential in causing HGSOC. This dualistic origin has been supported by a recent study that has demonstrated the development of HGSOC from both OSE and FTE cells. Tumors developed from both these sites differed in biological features such as latency, metastasis, gene expression, and response to the drugs [5]. Identifying the precise cell of origin is critical in early diagnosis, treatment, development of experimental models, and for identifying therapeutic targets.

In general, the number of genes that are mutated in sporadic HGSOC is low as compared to other tumor types. However, mutations in TP53 occur at nearly 100% frequency [6]. More than 59.1% of these mutations are missense [7], of which some of them were proven to be oncogenic and support tumorigenesis by promoting metastasis and resistance to chemotherapy drugs. Although mutations in the TP53 gene are prevalent, approaches to develop treatments have been thus far unsuccessful [8]. Mutations in other genes at a significant frequency are uncommon in HGSOC (except BRCA1 & BRCA2) to act as targets for developing drugs [9].

**Figure 1:**
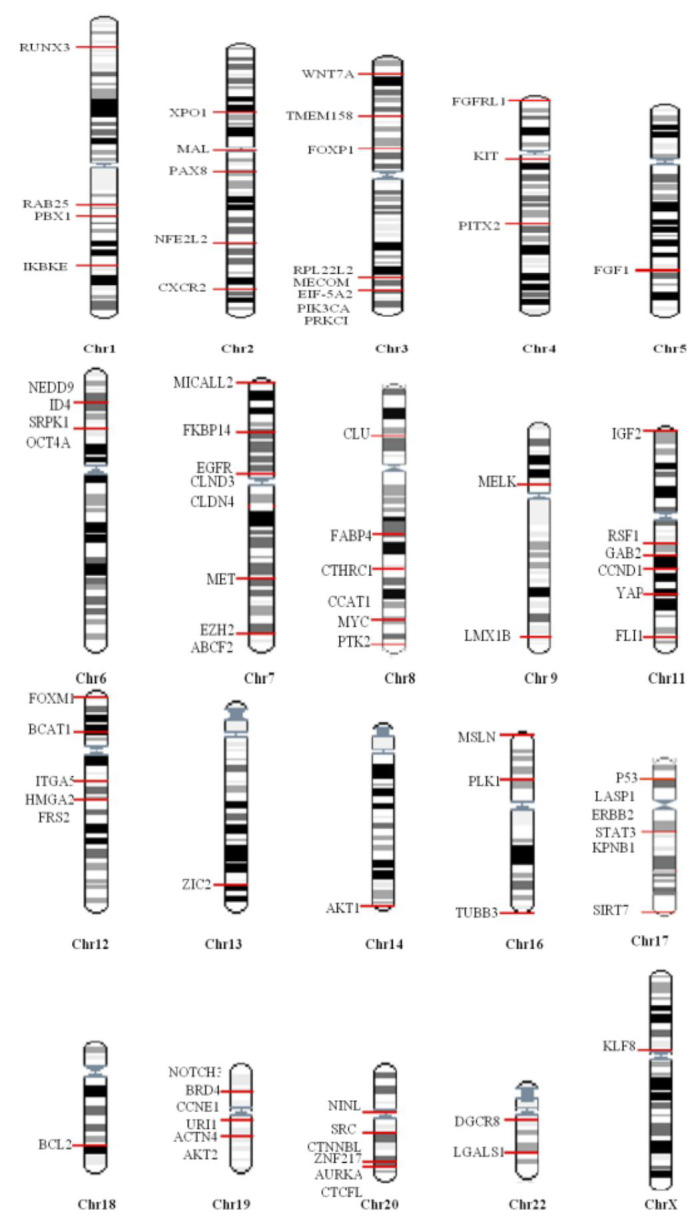
Schematic representation of the mechanism of alteration and characterization of oncogenes. Genetic and epigenetic alterations lead to the activation of oncogenes. The role of these activated oncogenes in the tumorigenesis can be studied by performing *in vitro* and *in vivo* studies. SCNA, Somatic Copy Number Amplification; TS miR, Tumor Suppressor microRNA; GEM, Genetically Engineered Mouse.

**Figure 2:**
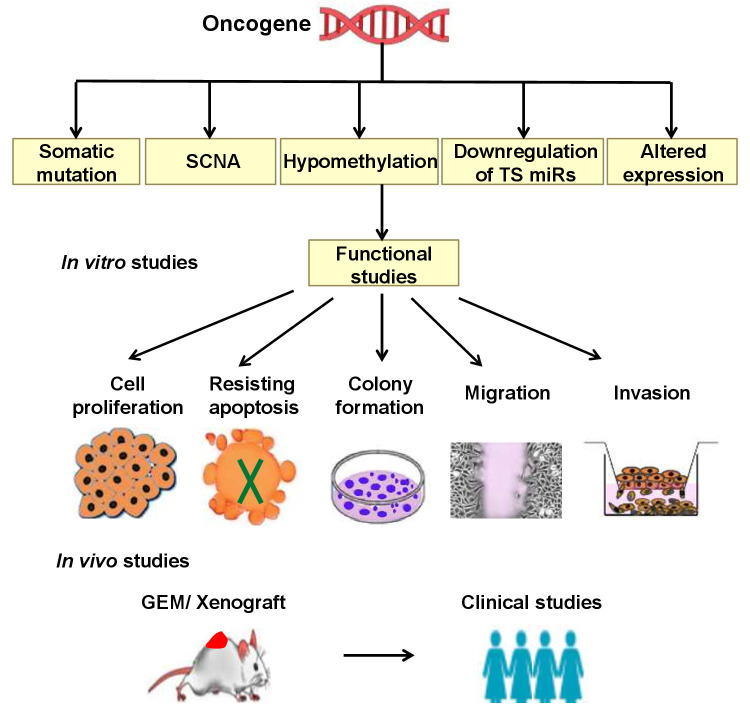
Chromosomal mapping of oncogenes in HGSOC. These genes were validated either by *in vitro* or *in vivo* methods.

Complex genetic changes with frequent DNA gains and losses are more common in HGSOC than in other tumor types. This leads to the activation of hundreds of oncogenes through gain and amplification, and inactivation of tumor suppressor’s through the loss of heterozygosity and homozygous deletion. Hence, it is challenging to identify essential cancer driver genes from the regions of amplification and deletion [9]. Both tumor suppressor genes and oncogenes are involved in multistep tumorigenesis. However, oncogenes are of particular interest as some are druggable [10]. Increased expression of any gene may be due to point mutation, amplification, increased transcription, hypomethylation, or as a result of a biallelic expression of imprinted genes [11]. Down-regulation of tumor suppressor micro RNA’s could also lead to the high expression of oncogenes [12] (Figure 1). Among these different genetic alterations, amplification is the most common mechanism promoting oncogene activation in HGSOC. The frequency with which other mechanisms occur is not clear, but mutations in genes other than TP53 are less frequent (<5%) [9]. Numerous oncogenes have been implicated in the pathogenesis of HGSOC (Figure 2), however, only some of them have been well characterized both *in vitro* and *in vivo*. Table 1 summarizes all the oncogenes that have been identified thus far and validated to be involved in the pathogenesis. These are not discussed in this review as they have been discussed previously [13]. The relevance of amplification/overexpression of the individual gene in HGSOC has been addressed by correlating it with the outcome. The evaluation of the role of these genes *in vitro* and *in vivo* also documented (Table 1).

Comprehensive genomic and epigenomic analysis of hundreds of tumors by international consortiums such as TCGA [9] and ICGC [14] has also led to a substantial increase in the discovery of these oncogenes. The contribution of these new genes to the pathogenesis of HGSOC is still being explored, and some of them appear to be attractive therapeutic targets [15]. Given the number of putative oncogenes identified in HGSOC, only further experiments will establish their role in tumorigenesis. Many of the oncogenes were previously identified by conventional gene transformation assay in NIH3T3 cells [16]. However, considering improvements in technology, it is now possible to perform large scale screening of the whole genome by functional approaches. This review discusses only the role of recently identified oncogenes in HGSOC. We particularly focus on oncogenes that were identified using strategies such as high throughput and genomic screening which were validated both *in vitro* and *in vivo*.

**Table 1a:** Table 1a: Oncogenes in HGSOC

**GENE**	**CHR**	**FREQUENCY OF ALTERATION**				**FUNCTIONS**		**OUTCOME**		REF
		**REPORT(N)**		**TCGA (N=316)**		***IN VITRO***	***IN VIVO***	**REPORT**	**TCGA**	
		**AMP**	**EXP (RNA/PTN)**	**AMP**	**EXP (RNA)**					
RAB25	1q22	54% (52)	88.7% (62)	6.9%	-	Cell proliferation, prevents apoptosis & anoikis	Ectopic expression induced tumors	↓ PFS & OS	NS	[84]
CXCR2	2q35	NA	69.2% (13)	0.3%	3.8%	Cell cycle progression & angiogenesis	Knockdown reduced tumor growth	↓ PFS & OS	NS	[85]
TMEM158	3q21.3	NA	84% (25)	1.58%	4.43%	Cell proliferation ,cell cycle progression, adhesion & invasion	Knockdown reduced tumor growth	NA	NS	[86]
RPL22L1	3q26.2	NA	NA	18.6%	3.4%	EMT, Invasion & metastasis	Ectopic expression induced tumors	NA	↑ OS	[87]
USP13	3q26.33	NA	NA	16.1%	6.32%	Cell proliferation & colony formation	Knockdown reduced tumor growth & metastatic nodules	↓ OS	NS	[88]
TRIM52	5q35.3	NA	90% (192)	1.26%	5.37%	Cell proliferation, migration, invasion & prevents apoptosis	Knockdown reduced tumor growth	NA	PFS	[89]
FABP4	8q21.13	NA	NA	1.2%	10%	Cell migration & invasion	Ectopic expression induced tumors & metastatic nodules	↓ PFS & OS	NS	[90]
YAP	11q22.1	NA	22.64% (106)	4.7%	4.4%	Cell proliferation, resistance, cell migration & anchorage-independent growth	Knockdown reduced tumor growth & metastatic nodules	↓ PFS	NS	[91]
URI1	19p12	9.4% (434)	34.9% (475)	17.4%	9.1%	Cell survival & cisplatin resistance	Knockdown reduced tumor growth	↓ PFS	OS	[92]
*NOTCH3*	19p13.12	NA	35.3% (44)	11.7%	4.1%	Cell proliferation, prevents apoptosis & anoikis	Knockdown reduced tumor growth & tumor nodules	NA	OS	[93]
ZNF217	20q13.2	59% (44)	NA	4.4%	1.5%	Proliferation & Metastasis	Ectopic expression induced tumors & metastasis	↓ PFS	NS	[94]
KLF8	Xp11.21	NA	76.3% (55)	0%		Transformation of Ovarian epithelial cells	Ectopic expression induced tumors	NA	-	[95]

The above mentioned genes are altered in ≥10% of patients with HGSOC

**Table 1b:** Table 1b: Oncogenes in HGSOC

**GENE**	**CHR**	**FREQUENCY OF ALTERATION**				**FUNCTIONS**		**OUTCOME**		REF
		**REPORT(N)**		**TCGA (N = 316)**		***IN VITRO***	***IN VIVO***	**REPORT**	**TCGA**	
		**AMP**	**OV EXP (RNA/PTN)**	**AMP**	**OV EXP (RNA)**					
FOXP1	3q13	NA	NA	1.9%	1.6%	Cancer stem cell properties ,spheroid formation, EMT, migration & chemoresistance	Knockdown reduced tumor growth	NA	NS	[96]
FGFRL1	4p16.3	NA	NA	1.9%	3.16%	Cell proliferation & migration	Knockdown reduced tumor growth	↓ OS	NS	[97]
PITX2	4q25	NA	NA	-	2.5%	Cell proliferation, anchorage independent growth & invasion	Ectopic expression induced tumors	NA	NS	[98]
OCT4A	6p21.33	NA	NA	2.5%	2.8%	Cell survival, metastasis & chemoresistance	Knockdown reduced tumor growth & metastasis	NA	NS	[99]
CTHRC1	8q22.3	NA	NA	8.8%	2.8%	EMT	Knockdown prevented the metastasis of tumors	↓ PFS & OS	NS	[100]
FOXM1	12p13.33	NA	NA	6%	4.1%	Cell proliferation, migration & invasion	Knockdown reduced the tumor growth,ascites& number of tumor nodules	NA	NS	[101]
ITGA5	12q13.13	NA	39% (107)	1.2%	5%	Cell adhesiion & invasion	Knockdown resulted in a decrease in the number of intra abdominal mets, reduced ascites formation, smaller tumor nodules & increased survival	↓PFS & OS	NS	[102]
STAT3	17q21.2	NA	NA	NA	0.9%	Migration& Invasion	Knockdown reduced tumor growth	NA	NS	[103]
DGCR8	22q11.21	NA	NA	0.9%	3.4%	Proliferation, migration & invasion	Knockdown reduced tumor growth	NA	NS	[104]

The above mentioned genes are altered in <10% of patients with HGSOC

Abbreviations: N, No. of Samples; CNA, Copy number array; EXP, Overexpression of the gene at RNA level; PTN, Protein; OS, overall survival; PFS, Progression free survival; NA, Not available; NS, Not significant.

Only three genes mentioned in the tables above were identified using different techniques; RAB25 (Comparative Genomic Hydribization), RPL22L1 (Degenerate oligonucleotide-primed polymerase chain reaction), and FABP4 (in silico analysis). Other genes are reported from publications.

The genes in italic font are druggable targets based on the data from IDG (Illuminating the Druggable Genome) database (https://commonfund.nih.gov/idg/index).

The information for a specific gene and its impact on survival of the patients with HGSOC (n=316) was determined using TCGA HGSOC data set (n=316) on cBioPortal

(https://www.cBioPortal.org/) The thresholds used in the analysis were: GENE NAME: AMP & EXP >=2

### Oncogenes identified by high-throughput functional screening

High-throughput functional assays are being widely used for discovering targets in cancer. This method permits the screening of hundreds of genes and identifies those that are important for a particular function. This is performed through gain of function studies i.e., through ectopic overexpression of selected genes using plasmids or by viral transduction into normal or cancer cell lines [17]. Besides, the role of the cancer-relevant genes can also be identified by perturbing the function of genes by small molecules such as chemical inhibitors, antibodies, RNA interference, or by CRISPR [18]. However, in both approaches, different assays are required to determine the effect of a particular gene. We discuss oncogenes identified using these approaches.

### PAX8 – Paired Box Gene 8 (2q14.1)

PAX8 is a nuclear transcription factor that belongs to the PAX (1-9) family of genes. It was initially discovered as one of the genes that are expressed and required for the development of thyroid. Later it was identified to play an essential role in the development of many organs, including Mullerian duct maturity which leads to the formation of the female genital tract. Hence homozygous deletion of this gene causes infertility in mice. Both PAX2 and PAX8 are necessary for the development of the fallopian tube [19]. Several studies have demonstrated that PAX8 is mainly expressed in the fallopian tube secretory epithelial cells (FTSEC), but absent in human ovarian surface epithelial cells (HOSE) [20] and mouse ovarian surface epithelial cells (MOSE) [21]. In contrast, others have shown that PAX8 is expressed both at the mRNA level (71%) and protein level (33%) in normal ovarian surface epithelium (OSE) [22]. Increased expression of PAX8 was also observed in cortical inclusion cysts and abnormal ovarian surface epithelial growths of the ovary [23]. Induced expression of PAX8 could not cause any malignant changes in transformed human OSE cells (expressing hTERT &c-MYC) [22]. In contrast, transfection of this gene in MOSE cells (transformed with myrAKT and PTEN deletion) resulted in high expression of FOXM1 and EMT markers causing increased cell proliferation and motility respectively but, failed to induce tumors [21]. Induced expression of PAX8 in these cells is alone not sufficient for promoting tumorigenesis.

High throughput shRNA screen performed by Project Achilles identified PAX8 as one of the potential oncogenes from 11,194 genes, which is necessary for the proliferation and survival of ovarian cancer cell lines (n = 25). PAX8 was overexpressed in the majority of the ovarian cancer cell lines. In HGSOC, this gene is amplified in 16% of patients [24] and the high expression of this gene is correlated with advanced tumor stage and decreased survival [25]. Knockdown of PAX8 in ovarian cancer cells with amplification or overexpression of this gene resulted in reduced viability, but not in cell lines without any alterations of this gene [24]. Silencing of PAX8 resulted in decreased migration, invasion, anchorage-independent growth, tumor formation, induced G1 arrest, and apoptosis in ovarian cancer cells. The molecular mechanism by which PAX8 promotes ovarian tumorigenesis is not completely understood. The current view is that PAX8 could directly bind to the promoter of mutant TP53 (missense mutation) in HGSOC cells and regulate its expression. Consequently, this results in overexpression and cytoplasmic translocation of the TP53 target gene p21 resulting in increased proliferation of HGSOC cells [19]. Cytoplasmic p21 has been proven to function as an oncogene by promoting the expression of anti-apoptotic genes and inhibiting pro-apoptotic genes [26]. Transfection of PAX8 into MOSE cells upregulates the expression of EMT markers via FOXM1 [21], a candidate oncogene in HGSOC (network altered in 84% of tumors) [9]. FOXM1 has been shown to promote the expression of EMT genes by binding to the promoter region in other tumor types [27]. PAX8 was shown to directly bind to the promoter regions of transcription factor E2F1 [19] and FGF18 promoting proliferation and migration respectively [28]. Both chromatin immunoprecipitation and expression analysis have identified that numerous oncogenes are regulated by PAX8 in HGSOC [19]. Although there are no PAX8-specific inhibitors, HDAC inhibitors (panobinostat & romidespin) were recently shown to effectively disrupt the expression and function of PAX8 both *in vitro* and *in vivo* [28]. Thus PAX8 seems to be an attractive target to develop drugs. In addition, the expression of PAX8 and PAX2 could be used to identify tumors of Mullerian origin [19].

### ID4 – Inhibitor of DNA binding 4 (6p22.3)

ID4 is a transcriptional regulator that contains the helix loop helix (HLH) domain. It interacts with basic helix loop helix (BHLH) domain-containing transcription factors inhibiting their binding to DNA. ID4 is necessary for the development of many organs, especially the ovaries. Mice lacking ID4 has increased secondary and antral follicles, altered ovary shape, decreased uterine weights, and estrogen synthesis [29]. The function of ID4 in different tumor types remains controversial. Increased hypermethylation and downregulation of this gene are associated with poor progression-free or overall survival in colon cancer [30], breast cancer [31], CLL [32], AML [33]. Whereas in glioblastoma and Estrogen Receptor (ER-) breast cancer ID4 was overexpressed [34].

An initial clue to the involvement of ID4 in ovarian cancer was documented through an inverse genomics study that identifies the function of a gene based on the phenotype observed upon altering the gene. This screen employed ribozymes to identify genes that regulate the expression of BRCA1. ID4 was identified as a negative regulator of the BRCA1 gene in PA-1 cells. Induced expression of ID4 in PA-1 cells promoted anchorage-independent growth [35]. Subsequently, ID4 was recognized as necessary for the proliferation and survival of ovarian cancer cells using data from Project Achilles. ID4 is amplified in 32% of 489 HGSOC tumors and is overexpressed in ovarian cancer cell lines and tumors, but is not expressed in normal ovary and fallopian tube [36]. Genetic alterations in this gene do not correlate with the survival of patients [7]. Silencing of ID4 in ovarian cancer cells reduced proliferation by inducing apoptosis. Induced expression of ID4 in immortalized ovarian surface epithelial cells (IOSE-M, expressing SV40 large T & small t antigens, hTERT, and MEKDD) resulted in increased colony formation and tumor growth in mice. In contrast, overexpression of ID4 in FTSEC-M cells caused only increased colony formation. The HLH domain of ID4 is necessary for the interaction with other oncogenes as mutations in this domain failed to induce tumors. Other members of the ID family (ID1-3) were unable to confer malignant changes upon overexpression. Ectopic transfection of ID4 in IOSE-M cells resulted in increased expression of HOXA family of genes [36], which are known for promoting HGSOC [37]. Silencing of HOXA9 in ID4 overexpressing IOSE-M cells resulted in inhibition of anchorage-independent growth, and tumor formation, but had a moderate effect on cell proliferation. These results suggest that HOXA9 is required for ID4 induced malignant transformation. Analysis of the TCGA HGSOC dataset demonstrated that patients with amplification of ID4 had high expression of genes that were downregulated by Tp53 & p21 [36]. These studies demonstrated the critical role of ID4 in HGSOC by negatively regulating the expression of BRCA1 and its ability to induce transformation in immortalized OSE cells [35, 36]. 

Mutant TP53 protein (R175H) has been shown to bind to the promoter of ID4 upon DNA damage in breast cancer cells [29]. It is worthwhile to analyze whether this mechanism occurs in HGSOC since 59.1% of patients have missense mutations in the TP53 gene [7].

### GAB2 - GRB2 associated binding protein 2 (11q14.1)

GAB2 functions as an adaptor molecule and is crucial for mediating protein-protein interactions. It functions downstream of the signaling pathway of RTK. It is considered as a key component in PI3K-AKT & ERK signaling pathways. Somatic amplification and overexpression of GAB2 have been noted in different tumor types [38].

GAB2 is frequently amplified in ovarian cancer and is mostly correlated with serous histology type [39]. According to the TCGA analysis, GAB2 is amplified in 44% of HGSOC tumors [40]. High expression of GAB2 correlates with better progression-free and overall survival in these patients, which is an unusual property for an oncogene [41]. GAB2 is upregulated in ovarian cancer cell lines compared to the ovary and HOSE cells. It promotes migration and invasion in ovarian cancer cells by enhancing the expression of EMT marker ZEB1 through activation of the PI3K pathway [42].

In addition to the above findings, the function of GAB2 in the pathogenesis of HGSOC was revealed by two large studies. A high-throughput siRNA loss of function screening assay, targeting 272 amplified genes in HGSOC and endometrioid ovarian tumor cell lines, identified GAB2 as being critically required for cell survival [43]. Further, the role of GAB2 in HGSOC was also identified by multiplexed stringent *in vivo* transformation screen. Genes amplified in HGSOC (n = 455) were transduced into immortalized HA1E-M cells and implanted in immunodeficient mice. Cells induced with GAB2 formed more tumors compared to other genes. Overexpression of GAB2 in immortalized cells (HA1E-M and IOSE) induced tumor formation, but in FTSEC it increased the number of colonies [40].

Knockdown of GAB2 resulted in reduced cell proliferation [40, 44], ascites induced cell migration [45], tumor growth, and formation of blood vessels. GAB2 mediates angiogenesis via overexpression of chemokines such as CXCL1, CXCL2 & CXCL8 that are dependent on the IKKβ pathway [44]. GAB2 performs all these various oncogenic functions by mediating PI3K/AKT1/mTOR, MAPK, and IKKβ pathways in tumor cells that harbor GAB2 alteration [40, 44]. Therefore GAB2 overexpressing tumors are more sensitive to inhibition by PI3K and mTOR inhibitors in conjunction with inhibitors of IKKβ in preclinical models [44]. The functional screens illustrate how GAB2 was proven to be an oncogene in HGSOC.

### BRD4 – Bromodomain 4 (19p13.12)

BRD4, a chromatin reader binds to acetylated histones which are transcribed and also to non-histone proteins. It also functions as a scaffold protein and transcription factor. BRD4 was initially found to be involved in the translocation t(15;19)(q13, p13.1) with the nuclear protein testis (NUT) gene in midline carcinomas. Following this report, BRD4 was demonstrated as an oncogene in many tumor types [46].

An invivo shRNA screen in OVCAR-8 cells directed against 800 druggable genes identified 40 genes that are crucial for survival, proliferation, and tumorigenicity in immune-compromised mice. BRD4 was identified as one of the essential genes and was evaluated in the pathogenesis of HGSOC as it could also be targeted by the inhibitor JQ1. JQ1 affects the binding of BRD4 to nuclear chromatin thereby influencing the transcription of many genes. Treatment with JQ1 effectively abrogated the growth of tumors in ovarian PDX models (patient-derived xenograft) overexpressing MYCN & c-Myc at high levels [15]. In contrast, another study showed that the treatment of ovarian cancer cell line (OVTOKO) with JQ1 for 24 hrs caused significant downregulation of FOXM1 and its transcriptional targets such as AURKB, Survivin CCNB1, and PLK1, whereas c-Myc was only transiently inhibited [47]. Silencing of BRD4 with shRNA or JQ1 in primary ovarian cancer cells led to reduced proliferation, colony formation [15, 47-49] cell cycle arrest at G0/G1, and suppressed tumor growth invivo [47, 49].

Recent evidence suggests that BRD4 could also contribute to the pathogenesis by regulating the transcription of genes relevant to cancer stem cells such as ALDH1A1, LIF, HES1, and WNT5A [50]. Further, BRD4 also interacts with immune checkpoint proteins such as PD-L1. Ovarian tumors with high expression of BRD4 are positively correlated with elevated expression of PD-L1. BRD4 was shown to bind directly to the PD-L1 gene and promotes its transcription. In murine xenograft, treatment with JQ1 significantly reduced the expression of PD-L1 on tumor and immune cells [51].

Although treatment of BRD4 overexpressing tumors with BET inhibitors was shown to suppress the growth, yet, some of the cancer cells develop resistance eventually through different mechanisms. Gene expression analysis of chronic JQ1 treated cells or resistant cells has identified the upregulation of numerous oncogenes. This includes EGR1, FOS, FGFR1-4, IGF1R, EGF1R, PRKCA, JAK1-3, EPHB3, ACVR1, ACVR2, TGFBR1, and CK1γ1. Hence, co-targeting the BRD4 overexpressing cells with BET and PI3K /AKT/MEK/ ERK/ /mTOR inhibitors have been shown to affect cell proliferation, survival, and tumor growth significantly through activation of apoptosis compared to JQ1 alone [48, 49]. Further, combined treatment using JQ1 and cisplatin suppressed the expression of ALDH1A1 and tumor growth efficiently in xenograft [50].

BRD4 is amplified in 19% of 599 HGSOC tumors and is associated with worse overall and progression-free survival [15]. Additionally, copy number analysis of tumors that lack BRCA is found to have amplification of the BRD4 gene. Hence BRD4 could serve as a target for these patients [52]. Silencing of BRD4 also increased the sensitivity of the tumor cells to CHEK1 [53] and PARP inhibitors [54]. Taken together, these studies suggest that aberrant expression of BRD4 promotes ovarian cancer progression and also immune evasion through increased PD-L1 expression. Several inhibitors of BRD4 proteins are being developed and many are in clinical trials for different tumor types. AZD-513 is a novel selective BET bromodomain inhibitor that recently entered phase I clinical trial for evaluation in ovarian tumors. Unlike JQ1, which binds monovalently to the protein, AZD-5153 binds in the bivalent mode. This results in a stronger displacement of the BRD4 from the chromatin at the lowest concentration of the inhibitor. Preclinically this drug has shown to be effective in BRD4 amplified HGSOC-PDX models [55].

### KPNB1 – Karyopherin beta-1 (17q21.32)

KPNB1 also known as importin beta is highly expressed during embryogenesis [56]. It functions primarily as a transporter of molecules with a nuclear localization signal (NLS) from the cytoplasm to the nucleus through the nuclear pore complex. KPNB1 associated with karyopherin alpha 2 (KPNA2) which together binds to the target molecule [57]. Some of the target genes of KPNB1 for nuclear localization are Cyclin E, Cyclin B1 [58], STAT3, NF-κB, Gli1, ERBB2, EGFR, C-Met, Death receptor 5 [59] Snail, Cathepsin L and Cux1 [60]. In addition, KPNB1 is also necessary for various cellular processes such as mitotic spindle assembly, nuclear pore, and nuclear membrane formation [61]. Overexpression of KPNB1 has been implicated in many tumor types and is associated with poor survival in patients with GBM [59] gastric cancer [62], and B cell lymphoma [63]. High expression of KPNA2, but not KPNB1 is associated with poor prognosis in patients with ovarian cancer [64, 65]. Analysis of the TCGA HGSOC dataset using cBioPortal shows that KPNB1 is not amplified at a higher frequency in these patients (8% out of 316 cases) [7], hence requires validation in a different cohort of patients.

KPNB1 was identified as an oncogene in ovarian cancer through an invivo shRNA screen. This screen was performed to identify novel druggable genes that were essential for tumor formation in ovarian cancer. In this study, shRNAs against 7490 genes were transduced into ovarian cancer cells and injected into mice. Sequencing for shRNAs that were depleted in the tumors, identified numerous essential genes. Additionally, a second CRISPR screen could also recapitulate the results from the shRNA screen. KPNB1, the second hit in these screens was evaluated further [65].

Functional experiments revealed that the silencing of KPNB1 resulted in cell cycle arrest since it modulates the expression of APC/C members (multiple anaphase-promoting complexes). Knockdown of KPNB1 reduced cell proliferation, tumor growth, and the number of tumor nodules in mice. The expression of tumor suppressor’s p21, p27, Bax and cleaved caspase-3 were marginally increased upon silencing KPNB1. The converse results were obtained when KPNB1 was overexpressed in ovarian cancer cells. Usage of two different importin inhibitors importazole and ivermectin prevented ovarian cancer cell proliferation in a dose-dependent manner. Ivermectin, an FDA approved anti-parasitic drug that blocks importin alpha/beta-induced multiphase cell cycle arrest and apoptosis in ovarian cancer cell lines. The expression of cell cycle and apoptosis-related genes were up-regulated following ivermectin treatment. Combination treatment with paclitaxel significantly decreased tumor growth and increased the expression of caspase 3/7 in comparison to the effect of the single-agent [65]. This study had proposed that the use of ivermectin along with chemotherapy drugs might provide better results. However, further research is necessary to identify whether KPNB1 mediates its effect through promoting nuclear transport of various oncogenes. Another uncertainty is that it is unclear what these inhibitors target, either KPNB1 alone or the other importin genes [66].

### Oncogenes identified by genomic approaches

The ability to perform the whole genome or exome sequencing of tumors by next-generation sequencing has contributed enormously to the identification of mutations in many genes. In addition, evaluation of other genetic aberrations such as copy number variations, methylation, mRNA and miRNA expression through different platforms has led to an exponential increase in the identification of several relevant genes in HGSOC where mutations are infrequent. By integrating these alterations with gene expression many driver genes were identified [9]. Two genes, LMX1b [67] and BCAT1 [68] identified recently through this approach.

### LMX1B- LIM homeobox transcription factor 1 beta (9q33.3)

Several genes that are critical for embryogenesis or normal development were identified as aberrantly expressed in many tumor types [69]. LMX1B is one among those whose oncogenic function has been identified in HGSOC [67]. This gene belongs to the LIM homeodomain-containing protein family that is critically required during body patterning and development of different organs. Haploinsufficiency of LMX1B causes a genetic abnormality known as Nail patella syndrome in humans. LMX1A (a member of LIM family) has shown to be expressed and required for the development ovarian stem cell niche of drosophila. Induced expression of LXM1B could rescue the signature of LMX1A loss-of-function. Thus both these genes could play a role in the development of ovary [70]. The role of LMX1B has not been thoroughly evaluated in any tumor type. Two studies have identified that this gene is methylated in prostate cancer [71] and leukemia [72]. However, there is no experimental evidence showing that this gene is downregulated and could function as a tumor suppressor gene in these tumor types. Comparative genomic hybridization analysis of mouse ovarian cancer cell lines (n = 10) that are deficient for TP53 or BRCA1 showed gain and amplification of chromosome 9q33.3 centered on the single gene LMX1B. High expression of LMX1B at mRNA levels was observed in 46% of (7/15) human ovarian cancer cell lines compared to normal OSE cell line T29. Increased expression of this gene was observed in primary tumors at both RNA and protein levels. High expression of LMX1B was associated with decreased overall survival in patients with ovarian cancer [67].

Functional studies revealed that the induced expression of LMX1B in both mouse and human ovarian cancer cell lines markedly increased the migration of the cells. However, there was no change in cell proliferation and colony formation ability. Mice injected with LMX1B overexpressing ovarian cancer cells promoted tumor formation. The converse results were observed when cells expressing LMX1B were silenced with shRNA. The overexpression of LMX1B in ovarian cancer cell lines increases the expression of NF-kB pathway members. Treatment of LMX1B overexpressing cells with NF-kB inhibitor resulted in decreased migratory ability [67]. Hence NF-kB pathway inhibitors might potentially affect LMX1B function in promoting ovarian cancer, which needs to be thoroughly evaluated.

### BCAT1-Branched Chain Amino acid Transaminase 1, cytosolic (12p12.1)

BCAT1 gene encodes for a cytosolic aminotransferase enzyme that is involved in the catabolism of essential branched amino acids [73]. BCAT1 was first identified to be amplified and overexpressed in undifferentiated mouse teratoma cell lines and was downregulated upon differentiation. c-Myc was found to transcriptionally regulate the expression of this gene via binding to its recognition sequences [74]. Several independent groups have demonstrated the role of BCAT1 in tumorigenesis. Overexpression of BCAT1 has shown to be associated with poor survival in patients with myeloid leukemia, GBM [73] colon [74], hepatocellular [75], urothelial [76], breast [77] and gastric cancer [78]. Notably, an increase in resistance to chemotherapeutic drugs was observed in tumors with high expression of BCAT1 [75, 77]. According to the TCGA data, BCAT1 is amplified in 16% of HGSOC’s and alterations in this gene do not correlate with the survival of patients [7].

In ovarian cancer, BCAT1 was first reported to be up-regulated in chemoresistant epithelial ovarian tumors, however, the mechanism of drug resistance was not identified [79]. Through a comprehensive methylation analysis between normal ovary, low grade, and HGSOC tumors, BCAT1 was identified as significantly hypomethylated in these tumors correlating with higher expression in tumors compared to the normal ovary. As observed in other tumor types, BCAT1 was shown to be regulated by c-Myc in ovarian cancer cells [68]. Silencing of BCAT1 arrested cells at S phase, significantly affected cell proliferation, colony formation, migration, invasive ability of ovarian cancer cells. However, there was no change in sensitivity to chemotherapeutic drugs [25]. Injection of BCAT1 silenced cells did not affect tumor size and volume of the ascites compared to the control. Only an increase in survival was observed in mice injected with BCAT1 silenced cells [68].

Microarray analysis of BCAT1 silenced cells demonstrated the downregulation of numerous genes involved in cell growth, proliferation, metabolism, and transcription. Since BCAT1 has a critical role in regulating metabolism, knockdown of this gene suppressed major metabolites like glycerophospholipids, sphingolipids, and genes involved in lipid metabolism, protein biosynthesis, of which IDH1/2, sulfotransferases and also-keto reductases AKR1C1, AKR1C2, AKR1C3 are known to be involved in tumorigenesis [68]. Other than hypomethylation, BCAT1 was identified as somatically amplified and overexpressed in ovarian cancer cell lines with high invasive and migratory ability [80].

These studies have shown the relevance of BCAT1 in the progression of HGSOC through altered metabolism. Silencing of BCAT1 in cells has not shown tumor regression of xenograft. The amplicon 12p12.1 contains six genes other than BCAT1 (KRAS, LRMP, CASC1, LYRM5, and IFLTD1 and C12orf77), of which KRAS is a known cancer gene [81]. Since the silencing of BCAT1 alone couldn’t suppress the tumor growth, the co-amplified genes might play a role in supporting tumorigenesis which needs further validation. This is an issue with both focal and arm level amplification of any chromosome. Unless all the genes that are mapped within an amplicon are analyzed it is difficult to attribute the contribution to one gene.

## DISCUSSION

Despite considerable effort to identify druggable driver genes in HGSOC, the yield thus far has been disappointing. This is partly due to the absence of a significant frequency of mutations in any gene other than TP53. To prove that any gene that is altered is involved in the pathogenesis of cancer requires a multi-pronged experimental approach. Dominantly acting genes or oncogenes are more likely to be involved if they are mutated at a significant frequency and if these alter its function. A classical example is the RAS oncogene which is mutated in 30% of tumors [82]. It is also necessary to prove conclusively that the gene is necessary for the initiation and sustaining of malignant transformation. If the putative oncogene is not mutated but amplified or over-expressed, then the level of proof that is required is of an order higher. CCNE1 is an example of an amplified oncogene in ovarian tumors [9]. The initial experiments to prove that a gene is involved in the pathogenesis should be by overexpressing the gene stably in normal ovarian surface or fallopian tube epithelial cells and then assess the effect on phenotype. This should evaluate the effect of overexpression on proliferation, anchorage, adhesion, apoptosis, migration, and invasion. Stable inducible expression of the gene is more stringent in interpreting these assays. Assuming that the gene is over-expressed or amplified at a sufficient level in ovarian cancer cell lines, knockdown experiments using CRISPR can be performed in them. The standard assay for tumourigenicity is to evaluate the ability of the putative oncogene to transform cells in nude mice. For any oncogene to be considered significant, its expression has to be evaluated in human tumor samples and correlated with outcome. For example, the expression of ERBB2 correlates adversely with outcome in breast cancer [83] It is now possible to address this issue immediately by examining the TCGA data initially and then confirming the results in a separate dataset. If a potential drug is available to inhibit the function of the putative oncogene it adds to the overall evidence. For example, BRD4 described in this review fulfills most of these criteria. However, with an increased understanding of the evolution and plasticity of the tumor genome, it is possible that what is the most relevant gene in a tumor at presentation may not be when it recurs in a patient. It is difficult to model this *in vitro* other than to evaluate the contribution of the putative oncogene in murine transplantation experiments. The recent approaches by large scale functional or sequencing approaches have identified various potential targets that are druggable and will help develop new approaches to treatment.

## Abbreviations

HGSOC: High Grade Serous Ovarian Carcinomas
OSE: Ovarian Surface Epithelium
CIC: Cortical Inclusion Cyst
TCGA: The Cancer Genome Atlas
ICGC: International Cancer Genome Consortium;
CRISPR: Clustered Regularly –Interspaced Short Palindromic Repeats
siRNA: small interfering Ribo Nucleic Acid
shRNA: short hairpin RiboNucleic Acid
HOSE: Human Ovarian Surface Epithelium
FTE: Fallopian Tube Epithelium
FTSEC: Fallopian Tube Secretory Epithelial Cells
MOSE: Mouse Ovarian Surface Epithelium
IOSE: Immortalized Ovarian Surface Epithelium
PARP: Poly (ADP-ribose) Polymerase
RTK: Receptor Tyrosine Kinase
EMT: Epithelial-Mesenchymal Transition
